# MRI-based response patterns during neoadjuvant chemotherapy can predict pathological (complete) response in patients with breast cancer

**DOI:** 10.1186/s13058-018-0950-x

**Published:** 2018-04-18

**Authors:** Briete Goorts, Kelly M. A. Dreuning, Janneke B. Houwers, Loes F. S. Kooreman, Evert-Jan G. Boerma, Ritse M. Mann, Marc B. I. Lobbes, Marjolein L. Smidt

**Affiliations:** 1 GROW - School for Oncology and Developmental Biology, Maastricht University Medical Center, Maastricht, the Netherlands; 2 Department of Surgery, Maastricht University Medical Center, Maastricht, the Netherlands; 3 Department of Radiology and Nuclear Medicine, Maastricht University Medical Center, P.O. Box 5800, 6202 AZ Maastricht, The Netherlands; 4 Department of Pathology, Maastricht University Medical Center, Maastricht, the Netherlands; 5 Department of Surgery, Zuyderland Medical Center, Sittard-Geleen, the Netherlands; 6 Department or Radiology and Nuclear Medicine, Radboud University Medical Center, Nijmegen, the Netherlands

**Keywords:** Breast Cancer, Neoadjuvant chemotherapy, Magnetic resonance imaging

## Abstract

**Background:**

The main purpose was to investigate the correlation between magnetic resonance imaging (MRI)-based response patterns halfway through neoadjuvant chemotherapy and immunotherapy (NAC) and pathological tumor response in patients with breast cancer. Secondary purposes were to compare the predictive value of MRI-based response patterns measured halfway through NAC and after NAC and to measure interobserver variability.

**Methods:**

All consecutive patients treated with NAC for primary invasive breast cancer from 2012 to 2015 and who underwent breast MRI before, halfway through (and after) NAC were included. All breast tumors were reassessed on MRI by two experienced breast radiologists and classified into six patterns: type 0 (complete radiologic response); type 1 (concentric shrinkage); type 2 (crumbling); type 3 (diffuse enhancement); type 4 (stable disease); type 5 (progressive disease). Percentages of tumors showing pathological complete response (pCR), > 50% tumor reduction and > 50% tumor diameter reduction per MRI-based response pattern were calculated. Correlation between MRI-based response patterns and pathological tumor reduction was studied with Pearson’s correlation coefficient, and interobserver agreement was tested with Cohen’s Kappa.

**Results:**

Patients (n = 76; mean age 53, range 29–72 years) with 80 tumors (4 bilateral) were included. There was significant correlation between these MRI-based response patterns halfway through NAC and tumor reduction on pathology assessment (reader 1 *r* = 0.33; *p* = 0.003 and reader 2 *r* = 0.45; *p* < 0.001). Type-0, type-1 or type-2 patterns halfway through NAC showed highest tumor reduction rates on pathology assessment, with > 50% tumor reduction in 90%, 78% and 65% of cases, respectively. In 83% of tumors with type 0 halfway through NAC, pathology assessment showed pCR. There was no significant correlation between MRI-based response patterns after NAC and tumor reduction rates on pathology assessment (reader 1 *r* = − 0.17; *p* = 0.145 and reader 2 *r* = − 0.17; *p* = 0.146). In 41% of tumors with type 0 after NAC, pathology assessment showed pCR.

**Conclusion:**

MRI-based response patterns halfway through NAC can predict pathologic response more accurately than MRI-based response patterns after NAC. Complete radiological response halfway NAC is associated with 83% pCR, while complete radiological response after NAC seems to be correct in only 41% of cases.

**Electronic supplementary material:**

The online version of this article (10.1186/s13058-018-0950-x) contains supplementary material, which is available to authorized users.

## Background

neoadjuvant chemotherapy and immunotherapy (NAC) is considered the standard regimen for patients with locally advanced breast cancer and is increasingly being used in patients with early-stage breast cancer. Its main goal is to decrease tumor size [1]. By decreasing tumor size, NAC may enable patients, who would otherwise undergo mastectomy, to be treated with breast-conserving therapy. In patients initially scheduled for breast-conserving therapy a smaller lumpectomy might be performed, potentially resulting in improved cosmesis. Furthermore, NAC allows in vivo assessment of tumor response and therefore chemosensitivity. Individual responses to NAC vary widely, depending on molecular subtype (*i.e.* estrogen receptor (ER) negative and human epidermal growth factor receptor 2 (HER2) positive tumors respond better) [2], tumor size [3] and treatment regimen [4–6].

In order to achieve the maximum surgical advantage from NAC, it is essential that tumor response and residual tumor can be evaluated correctly prior to surgery. Studies show that magnetic resonance imaging (MRI) is the most accurate in determining residual disease after NAC compared to physical examination, mammography and ultrasound [7]. Unfortunately, MRI might both overestimate or underestimate residual tumor size. The overall loss of vital tumor cells might not always be reflected by a reduction in tumor diameter as fibrous stroma might persist and even be enhanced on MRI [8]. Tumor size assessment itself on MRI might also be challenging, as NAC causes various histopathological changes in tumor cellularity, causing some tumors to show concentric shrinkage patterns, while others may crumble (“fragmentation”) into scattered islands of tumor cells. In the latter case, a response is present, but this might not be expressed in simply measuring tumor size, as these individual scattered foci cannot be measured independently on MRI.

Previous research shows that triple negative breast tumors regress significantly more often as a shrinking mass than HER2 positive and ER positive/HER2 negative tumors [9]. In addition, Kim et al. demonstrated that there is a significant difference in MRI-based response patterns after NAC between pathological responders and non-responders [8]. However, these studies analyzed response patterns after completion of NAC. At this point, changes in treatment regimen are no longer possible. Furthermore, the ACRIN study suggests that MRI early in NAC treatment is a stronger predictor of pathological response than MRI after NAC [10]. Hence, studying the association between response patterns on MRI during NAC and final histopathological response (when switching to a cross-resistant NAC in cases with poor (predicted) response might still be possible) could have more clinical implications. To our knowledge, no study has tested correlation between MRI-based response patterns halfway through NAC and pathological response.

Therefore, the main goal of this study was to analyze MRI-based response patterns halfway through NAC and to investigate their role as an early therapy response predictor. Secondary goals were to compare the predictive value of MRI-based response patterns measured halfway through NAC to after NAC and to compare preoperative tumor diameter on breast MRI with tumor size on pathology assessment to evaluate residual tumor assessment in different MRI-based response patterns. Finally, we evaluated interobserver agreement for assessment of MRI-based response patterns and tumor diameter.

## Methods

### Patient selection

We included all consecutive patients who were treated with NAC for histologically proven primary invasive breast cancer between January 2012 and June 2015, and in whom tumor response was monitored with MRI in the Maastricht University Medical Center+ (MUMC+). In this hospital, standard response monitoring with MRI was performed before, halfway through and after NAC. MRI after NAC was often not performed in the case of complete radiological response halfway through NAC or in the case of mastectomy. Patients who underwent surgery after NAC and underwent at least two MRI examinations were included in this study, provided that the first MRI was performed at baseline, *i.e.* prior to NAC, and the second MRI after completion of at least three cycles of chemotherapy. Exclusion criteria were unknown ER, progesterone receptor (PR), or HER2 status prior to NAC, previous ipsilateral breast surgery, previous systemic treatment because of contralateral breast cancer and presence of distant metastasis at time of diagnosis.

### MRI protocol

Breast MRI was performed on a 1.5 T scanner using a dedicated bilateral 16-channel breast coil (Philips Healthcare, Best, The Netherlands). As contrast agent, gadobutrol (Gadovist®, Bayer Health Care, Germany) was automatically injected through a catheter in the antecubital vein at 0.1 mmol/kg body weight, followed by a saline flush. The imaging aprotocol consisted of two-dimensional T2-weighted images without fat suppresion, dynamic contrast-enhanced fat-saturated T1-weighted images using gadobutrol as a contrast agent, and diffusion weighted imaging (DWI). Imaging parameters can be found in Additional file 1.

### Imaging analysis and tumor response pattern assessment on MRI

Two experienced breast radiologists (JBH and RMM), with 7 and 12 years of experience respectively, independently reviewed all breast MRI scans. They were blinded to pathological tumor characteristics and pathological outcome after surgery.

**Fig. 1:**
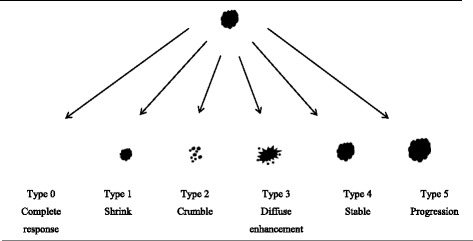
Magnetic resonance imaging (MRI)-based response patterns of breast carcinomas on breast MRI halfway through and after neoadjuvant chemotherapy

MRI-based response patterns of breast carcinomas during and after NAC were classified into six categories adapted from the classification suggested by Kim et al. [8] (Fig. 1): type 0 (complete radiologic response); type 2 (concentric shrinkage > 3 mm without surrounding lesions); type 2 (crumbling: shrinkage with residual multinodular lesions); type 3 (diffuse contrast enhancement in whole quadrants); type 4 (stable disease, i.e. no response, shrinkage < 3 mm or increase < 3 mm); type 5 (progressive disease, i.e. increase in tumor size > 3 mm or new lesions). A short introduction about the MRI-based response patterns and a test case were provided for both readers. Furthermore, tumor size was determined by measuring the largest diameter of the largest breast lesion on the T1-weighted MRI sequence at peak enhancement (*i.e.* first dynamic phase after contrast injection) in one view. Readers were allowed to use multiplanar reconstructions to assess the largest tumor diameter.

### Treatment

All patients received systemic treatment and underwent surgery at the MUMC+. NAC treatment consisted of two possible regimens. All patients with HER2-negative tumors received six cycles of docetaxel, doxorubicin and cyclophosphamide. HER2-positive tumors were treated with four cycles of doxorubicin and cyclophosphamide followed by four cycles of docetaxel and trastuzumab. After NAC, breast conserving therapy or mastectomy and surgery of the ipsilateral axilla (sentinel lymph node biopsy in the case of node-negative (N0) and axillary lymph node dissection in the case of N+) were performed.

### Histopathological assessment

All pre-treatment core biopsies and post-treatment surgical specimens were routinely processed. Histopathological analyses were performed by an experienced breast pathologist in accordance with our national breast cancer guideline at the time of diagnosis [11].

Pre-treatment core biopsies were used for grading (according to the modified Bloom-Richardson grading system) and determining receptor status of the tumor. Hormone receptor status, *i.e.* ER and PR status, was determined by immunohistochemical evaluation and interpreted according to national guidelines in which > 10% of tumor staining is used as a positive cutoff. HER2 status was determined using fluorescence in situ hybridization (FISH) analysis to detect gene amplification in biopsied tissue and was analyzed according to the ASCO CAP guidelines [12]. Tumors were stratified into molecular subtypes based on immunohistochemical evaluation and FISH. Hormone receptor status was considered positive (ER+) when ER and/or PR status was positive and negative (ER-) if both were negative. There were four molecular subtypes: ER+/HER2-, ER+/HER2+, ER-/HER2+ and ER-/HER2-.

Histopathological measurement of residual tumor size, which is considered to be the gold standard, was performed in fresh tissue and correlation was tested microscopically in formalin-fixed, paraffin-embedded tissue. This assessment only included invasive foci, not ductal carcinoma in situ (DCIS). DCIS was measured separately. Pathological dimensions were determined using the longest diameter of the residual tumor or in the case of multifocal disease, the primary index tumor. Thereafter, specimens were fixed with formalin.

Histopathological response of the tumor to treatment was evaluated based on reduction of tumor cellularity, using the Pinder classification (Table 1). Pathological complete response (pCR) was defined as absence of macroscopic and microscopic evidence of invasive tumor and absence of ductal carcinoma in situ (DCIS).

**Table 1:** Pinder classification

Pinder classification	Explanation
1i	Pathological complete response, no DCIS
1ii	Pathological complete response, including DCIS
2i	Response > 90% (or < 10% invasive tumor left)
2ii	Response of 50–90% (or 10–50% invasive tumor left)
2iii	Response < 50% (or > 0% invasive tumor left)
3	No signs of response

*DCIS* ductal carcinoma in situ

For this study, patients with Pinder classification 2iii or 3 (*i.e.* < 50% or no regression in tumor cells) were categorized as non-responders, while patients with Pinder classification 1i–2ii (*i.e.* ≥ 50% regression in tumor cells) were classified as pathological responders.

### Statistical analysis

Pearson’s correlation coefficient was used to test correlation between the MRI-based response patterns (halfway through and after NAC) and pathological response after NAC. In case multifocal disease was present, the lesion with the largest dimensions on baseline MRI, considered to be the primary index tumor, was included for statistical analysis. Interobserver agreement between both readers classifying the response according to the six MRI-based patterns was calculated with Cohen’s Kappa [13]. The distribution of the MRI-based response patterns in the different breast cancer subtypes was mapped. Mean tumor size and agreement between both readers was calculated. Pearson’s correlation coefficient was also used to test correlation between tumor size on MRI after NAC and pathologically assessed tumor size (gold standard). When a difference of more than 5 mm between both measurement techniques or between both readers was observed, this was considered to be clinically relevant. A *p* value <0.05 was considered to be statistically significant. All analyses were performed using Statistical Package for the Social Sciences (SPSS), version 22.0 (IBM Corporation, Armonk, NY, USA).

## Results

A total of 76 patients with 80 primary breast tumors (4 bilateral) were included. All patients underwent a breast MRI exam before and halfway through NAC; 57 patients also underwent a breast MRI exam after completion of NAC. Baseline characteristics are shown in Table 2. Mean age was 53 years (range 29–72). Most tumors (89%) were classified as invasive carcinoma of no special type (NST) and 11% were lobular carcinomas. Considering receptor status, most tumors (60%) were ER/PR positive and HER2 negative (Table 2).

**Table 2:** Baseline characteristics

Characteristic	Number		Percentage
Age, years			
Mean (*n* = 76)		53	
Range		29–72	
T-stage prior to NAC			
T1	13		16
T2	46		58
T3	15		19
T4	5		6
Tx	1		1
N-stage prior to NAC			
N0	49		61
N1	22		28
N2	3		4
N3	6		8
Histology			
Invasive carcinoma NST	71		89
Lobular carcinoma	9		11
DCIS			
Present	21		26
Absent	45		56
Unknown	14		18
Subtype by receptor status			
ER/PR+ HER2+	13		16
ER/PR+ HER2-	48		60
ER- PR-HER2+	6		8
ER-PR-HER2-	13		16
Grade			
1	2		3
2	35		44
3	25		31
Unknown	18		23
Surgical method			
Mastectomy	46		58
Lumpectomy	34		43

*Abbreviations*: *T-stage* tumor stage, *NAC* neoadjuvant chemotherapy and immunotherapy, *N-stage* nodal stage, *NST* no special type, *DCIS* ductal carcinoma in situ, *ER* estrogen receptor, *PR* progesterone receptor, *HER2* human epidermal growth factor receptor 2

In ten tumors pathological tumor response was assessed with the Miller and Payne grading system and in five of them this classification could not be converted to the Pinder classification. Therefore, these five patients could not be included in any pathological response analyses, but were included in the evaluation of residual tumor size. Furthermore, since reader 1 did not classify any tumors as showing diffuse enhancement (type 3), most analysis did not include the type 3 response pattern.

### Baseline MRI - tumor diameter and interobserver agreement

Mean baseline tumor diameter was 30 mm (range 11–76 mm) according to reader 1 and 32 mm (range 12–118 mm) according to reader 2. At baseline tumor diameter differed more than 5 mm between both readers in 28 tumors (35%), but in only 5 of them (6%) this difference resulted in a difference in clinical tumor stage

### MRI halfway through NAC - response patterns and interobserver agreement

**Fig. 2:**
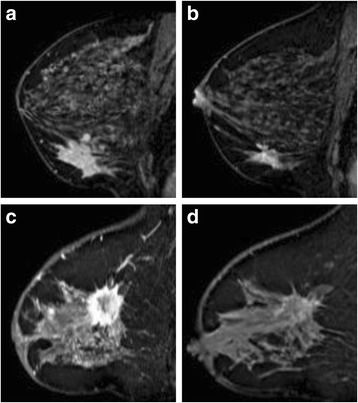
Example of a tumor that shrinks concentrically: magnetic resonance imaging (MRI) before neoadjuvant chemotherapy and immunotherapy (NAC) (**a**) and halfway through NAC (**b**); and a tumor that crumbles: MRI before NAC (**c**) and halfway through NAC (**d**)

Figure 2 shows an example of a type 1 response (concentrically shrinking tumor) and of a type 2 response (crumbling tumor).

As shown in Tables 3 and 5, tumors showing a type-0 response (complete radiologic response) on MRI halfway through NAC have the best pathologic tumor response (90% had > 50% tumor reduction and 83% had pCR).

**Table 3:** MRI-based response patterns of breast carcinomas on breast MRI halfway through NAC and pathological response per MRI-based response pattern

	Reader 1				Reader 2			
	Number (percentage)	Tumor response > 50%	Diameter reduction > 50%	pCR	Number (percentage)	Tumor response > 50%	Diameter reduction > 50%	pCR
Complete radiologic response (type 0)	5 (7%)	4/5 (80%)	4/5 (80%)	4/5 (80%)	7 (9%)	7/7 (100%)	6/7 (86%)	6/7 (86%)
Concentric shrinkage (type 1)	38 (51%)	30/38 (79%)	14/38 (37%)	9/38 (24%)	22 (29%)	17/22 (77%)	8/22 (36%)	6/22 (27%)
Crumbling (type 2)	21 (28%)	13/21 (62%)	8/19^a^ (42%)	3/21 (14%)	24 (32%)	16/24 (67%)	9/24 (38%)	3/24 (13%)
Diffuse enhancement (type 3)	0 (0%)	n.a.	n.a.	n.a.	3 (4%)	3/3 (100%)	0/2^a^ (0%)	0/3 (0%)
Stable disease (type 4)	11 (15%)	5/11 (55%)	4/11 (36%)	0/11 (0%)	18 (27%)	9/18 (50%)	7/17^a^ (41%)	1/18 (6%)
Progressive disease (type 5)	0 (0%)	n.a.	n.a.	n.a.	1 (1%)	0/1 (0%)	0/1 (0%)	0/1 (0%)
Total	75 (100%)	52/75 (69%)	30/73^a^ (41%)	16/75 (21%)	75 (100%)	52/75 (69%)	30/73^a^ (41%)	16/75 (21%)

*Abbreviations: MRI magnetic resonance imaging, pCR* pathological complete response, *n.a.* not applicable

^a^In two tumors no diameter assessment was possible on baseline MRI

Patients with a type-2 response (crumbling tumors) less often had > 50% tumor reduction (65% vs 78%) and less often had pCR (14% vs 26%) than patients with tumors with a type 1 response (concentric shrinking) halfway through NAC. Similar rates were seen for tumor diameter reduction > 50% in patients with type-1 and a type-2 response halfway through NAC (40% vs 37%).

There was weak but significant correlation between the MRI-based response patterns halfway through NAC and pathological tumor reduction; lower MRI response patterns (type 0–2) were related to higher pathological tumor reduction rates than higher MRI response patterns (type 3–5) (*r* = 0.33; *p* = 0.003 for reader 1 and *r* = 0.445; *p* < 0.001 for reader 2).

Among patients with pCR, nearly all tumors with pCR (*n* = 16 (20%)) showed a type 0, 1 or 2 response on MRI halfway through NAC (except for one tumor classified as type 4 by reader 2). Considering interobserver agreement, in 40/80 cases (50%) both readers classified the tumor into the same MRI-based response pattern halfway through NAC; in the other half of the cases they disagreed. Interobserver agreement between reader 1 and 2 was therefore considered fair (κ = 0.301).

Halfway through NAC mean tumor sizes determined on MRI by readers 1 and 2 were 19 mm (range 0–54 mm) and 24 mm (range 0–119 mm), respectively. Halfway through NAC tumor there were diameter differences > 5 mm between the two readers’ assessments in 25 patients (31%). Most differences in tumor diameter were in tumors classified as crumbling (58%) and occurred in around 25% (21–33%) of tumors with the other MRI-response patterns.

### MRI after NAC - response patterns and interobserver agreement

As shown in Tables 4 and 5, tumors showing a type-0 response (complete response) after NAC had the best pathological tumor response (96% had > 50% tumor reduction and 41% had pCR) followed by both the type 2 (crumbling) and the type 1 response (concentric shrinking tumors), which seem to downsize at similar rates on pathological assessmenbt (Tables 4 and 5).

**Table 4:** MRI-based response patterns of breast carcinomas on breast MRI after NAC and pathological response per MRI-based response pattern

	Reader 1				Reader 2			
	Number (percentage)	Tumor response > 50%	Diameter reduction > 50%	pCR	Number (percentage)	Tumor response > 50%	Diameter reduction > 50%	pCR
Complete radiologic response (type 0)	13 (23%)	12/13 (92%)	7/12^a^ (58%)	5/13 (38%)	9 (16%)	9/9 (100%)	5/8^a^ (63%)	4/9 (44%)
Concentric shrinkage (type 1)	19 (33%)	11/19 (58%)	7/19 (37%)	2/19 (11%)	8 (14%)	5/8 (63%)	4/8 (50%)	2/8 (25%)
Crumbling (type 2)	11 (19%)	7/11 (64%)	5/11 (45%)	1/11 (9%)	15 (26%)	11/15 (73%)	6/15 (40%)	1/15 (6%)
Diffuse enhancement (type 3)	0 (0%)	n.a.	n.a.	n.a.	10 (18%)	8/10 (80%)	5/10 (50%)	2/10 (20%)
Stable disease (type 4)	13 (23%)	6/13 (46%)	2/13 (15%)	1/13 (8%)	14 (25%)	4/14 (29%)	1/14 (7%)	0/14 (0%)
Progressive disease (type 5)	1 (2%)	1/1 (100%)	0/1 (0%)	0/1 (0%)	1 (2%)	0/1 (0%)	0/1 (0%)	0/1 (0%)
Total	57 (100%)	37/57 (65%)	21/56^a^ (38%)	9/57 (16%)	57 (100%)	37/57 (65%)	21/56^a^ (38%)	9/57 (16%)

*Abbreviations: MRI magnetic resonance imaging, NAC neoadjuvant chemotherapy and immunotherapy, pCR* pathological complete response

^a^In one tumor no diameter assessment was possible on baseline MRI

**Table 5:** Mean percentages of pathological response per MRI-based response pattern (mean percentage of reader 1 and 2) halfway through and after NAC

	MRI halfway through NAC				MRI after NAC			
	Percentage of total (*N* = 75)	Tumor response > 50%	Diameter reduction > 50%	pCR	Percentage of total (*N* = 57)	Tumor response > 50%	Diameter reduction > 50%	pCR
Complete radiologic response (type 0)	8%	90%	83%	83%	20%	96%	61%	41%
Concentric shrinkage (type 1)	40%	78%	37%	26%	24%	61%	44%	18%
Crumbling (type 2)	30%	65%	40%	14%	23%	69%	43%	8%
Diffuse enhancement (type 3)	2%	n.a.	n.a.	n.a.	9%	n.a.	n.a.	n.a.
Stable disease (type 4)	21%	53%	39%	3%	24%	38%	11%	4%
Progressive disease (type 5)	1%	n.a.	n.a.	n.a.	2%	50%	0%	0%
Total	100%	69%	41%	21%	100%	65%	38%	16%

*Abbreviations: MRI* magnetic resonance imaging, *NAC* neoadjuvant chemotherapy and immunotherapy, *pCR* pathological complete response, *n.a.* not applicable

When we look further into the type-0 response after NAC, we see that 10 out of 16 tumors classified as having a type-0 response (complete radiological response) by one or both readers on MRI after NAC still showed residual tumor on pathological assessment. None of these 10 tumors were triple negative and 2 were lobular carcinomas.

There was no significant correlation between the MRI-based response patterns after NAC and pathological response after NAC (*r* = − 0.170; *p* = 0.145 for reader 1 and *r* = − 0.169; *p* = 0.146 for reader 2). Among patients with pCR, we observed that out of 16 patients with pCR, 7 did not undergo MRI after NAC: out of the 9 patients that did, 4 was classified as type 0 by both readers (complete response, true negatives), another was classified as type 0 by reader 1 but as type 3 by reader 2. The other four patients were classified as a type-1 or type-2 response and one was even classified as having type-4 (reader 1) and type-3 (reader 2) responses.

Considering interobserver agreement, in 27/57 cases (47%) both readers classified the tumor into the same MRI-based response pattern halfway through NAC. In the other 53% they disagreed. Interobserver agreement between reader 1 and 2 was therefore also considered fair (κ = 0.312).

The mean residual tumor sizes on MRI according to readers 1 and 2 were 13 mm (range 0–50 mm) and 23 mm (range 0–105 mm), respectively. Mean pathological tumor size after NAC was 21 mm (range 0–105 mm). Tumor diameter after NAC differed by more than 5 mm between the two readers in 22 patients (39%). Tumor diameter differences between readers were least common in complete radiological responders (20%) and were observed in 35%, 40%, 47% and 75% of patients with types 1, 2, 4 and 5 MRI-response patterns, respectively.

Furthermore, tumor diameter differed by more than 5 mm between reader 1 and pathological assessment in 35 tumors (61%, of which size was underestimated in 66%) and between reader 2 and pathological assessment in 33 tumors (58%, of which size was underestimated in 42%). For reader 1, most differences in measurement were in tumors classified as concentric shrinking tumors (70%) followed by the crumbling tumors (64%). For reader 2, most of the differences in measurement were in tumors classified as diffuse enhancing tumors (78%) followed by concentric shrinking (63%) and crumbling (53%) tumors.

### Surgery and MRI-based response patterns

Mastectomy was performed in 46 patients and breast conserving therapy in 34 patients. In 7/80 patients the tumor was not completely removed. Characteristics of these seven tumors are displayed in Table 6. There was no significant correlation between MRI-based response patterns halfway through or after NAC and incomplete resection (even though types 1 and 4 seem to predominate). In two of these tumors, the tumor diameter was underestimated by more than 5 mm by both readers, and was underestimated in three tumors by one reader.

**Table 6:** Characteristics of incompletely removed tumors (tumor-positive margins)

	Tumor type	MRI-pattern halfway through NAC (R1)	MRI-pattern halfway through NAC (R2)	MRI pattern after NAC (R1)	MRI pattern after NAC (R2)	Tumor size after NAC (R1)	Tumor size after NAC (R2)	Surgery	Pathological tumor size
1	Ductal	1	1	1	1	18	23	Lumpectomy	19
2	Lobular	0	2	MRI not performed	MRI not performed	MRI not performed	MRI not performed	Mastectomy	105
3	Ductal	1	1	1	3	4	22	Lumpectomy	17
4	Ductal	2	4	4	4	20	15	Mastectomy	80
5	Ductal	1	2	1	4	23	20	Lumpectomy	40
6	Ductal	1	1	1	4	8	15	Lumpectomy	17
7	Ductal	1	4	4	4	6	17	Lumpectomy	21

*Abbreviations*: *MRI*, magnetic resonance imaging, *NAC* neoadjuvant chemotherapy and immunotherapy, *R1* reader 1, *R2* reader 2

### Subtype and MRI-based response patterns

As shown in Table 7, 16% of tumors were classified as ER + HER2+, 60% as ER + HER2-, 8% as ER-HER2+ and 16% as ER-HER2-. Numbers were too small for subtype analyses but we observed that only type 0 or 1 MRI patterns halfway through NAC led to pCR for ER + HER2- tumors while in other subtypes a type 2 (or exceptionally type 4) tumor could become classified as pCR halfway through NAC.

**Table 7:** Number of tumors per subtype, number of tumors per subtype with pathological complete response and their MRI-based response patterns

Subtype	Number of tumors (percentage)	Number with pCR (percentage)	MRI patterns halfway through NAC in tumors showing pCR (R1 and R2)	MRI patterns after NAC in tumors showing pCR (R1 and R2)
ER+/HER2+	13 (16%)	3 (23%)	0, 1, 2, 4	0, 1, 2
ER+/HER2-	48 (60%)	4 (8%)	0, 1	0, 1
ER-/HER2+	6 (8%)	3 (50%)	0, 1, 2	0, 1, 2
ER-/HER2-	13 (16%)	6 (46%)	0, 1, 2	0, 3, 4
Total	80 (100%)	16 (20%)	0, 1, 2, 4	0, 1, 2, 3, 4

*Abbreviations*: *MRI* magnetic resonance imaging, *pCR* pathological complete response, *NAC* neoadjuvant chemotherapy and immunotherapy, *ER* estrogen receptor, *HER2* human epidermal growth factor receptor 2*, R1* reader 1, *R2* reader 2

## Discussion

One of the main reasons for starting neoadjuvant chemotherapy in patients with breast cancer is to decrease tumor volume. Individual responses to NAC vary, depending on molecular subtype, tumor size and treatment regimen, but also depending on factors that are still unknown. Predicting individual response to NAC remains difficult. Enabling response prediction during NAC would help determine the usefulness of NAC and may lead to alterations in treatment regimen or performing surgery earlier than initially planned. To further explore the possibilities of response prediction, the main goal of this study was to investigate the correlation between six MRI-based response patterns halfway through NAC and pathological evidence of tumor response.

Secondary goals were to compare the predictive value of MRI-based response patterns measured halfway through to after NAC and to evaluate interobserver agreement. Furthermore, to achieve the maximum surgical advantage from NAC, it is essential that tumor response and residual tumor are assessed correctly before surgery. In this study, we compared preoperative tumor diameter on MRI with pathological tumor size and evaluated the assessment of residual tumor in different MRI-based response patterns after NAC.

In this study, there was significant correlation between MRI-based response patterns measured halfway through NAC and pathological evidence of tumor reduction (*r* = 0.33; *p* = 0.003 for reader 1 and *r* = 0.45; *p* < 0.001 for reader 2). Tumors with a type-0 response (complete radiological response) halfway through NAC had a 90% chance of pathological evidence of tumor reduction > 50% and 83% chance of pCR. Tumors with a type-2 response (crumbling tumors) less often had tumor reduction > 50% (65% vs 78%) and less often had pCR (14% vs 26%) than tumors with a type-1 response (concentric shrinking) halfway through NAC. Tumors with a type-4 (stable disease) or type-5 response (progression) halfway through NAC had the lowest chances of tumor regression. A tumor with a type-4 response (stable disease) halfway through NAC only had a 3% chance of being classified as pCR.

Since nearly all tumors with pCR (*n* = 16 (20%)) had a type 0, 1 or 2 response halfway through NAC (except for one tumor classified as type 4 by reader 2), we can conclude that for a tumor to have pCR, a type 0, 1 or 2 response is required halfway through NAC.

There was no correlation between MRI-based response patterns measured after NAC (prior to surgery) and pathological evidence of tumor reduction (*r* = − 0.170; *p* = 0.145 for reader 1 and *r* = − 0.169; *p* = 0.146 for reader 2). The greatest pathological tumor reduction was observed in tumors with a type-0 response after NAC (96% had tumor reduction > 50% and 41% had pCR) followed by type 1 (concentric shrinking) (61% had tumor reduction > 50% and had 18% pCR) and type 2 response (crumbling tumors) (69% had tumor reduction > 50% and 8% had pCR). This implies that even if the radiologist no longer detects suspicious lesions (type 0) on MRI after NAC, around 60% of the patients still have invasive carcinoma or DCIS on pathological examination. In conclusion, as we hypothesized, the MRI-based response patterns measured halfway through NAC correlate better with pathological tumor response than MRI-based response patterns measured after NAC.

In our study both radiologists performed an independent reading and we observed fair interobserver agreement halfway through and after NAC, for both the classification of MRI-based response patterns and for tumor diameter measurements. Only half of the tumors were classified as the same MRI-based response pattern, and tumor diameter differences >5 mm occurred in 31–61% of cases, consisting of both underestimation and overestimation of size, and occurring in all MRI-based response patterns. Nevertheless, both readers’ MRI-based response classifications correlated similarly with pathological response. Only in 7/80 patients was the tumor not completely removed (and 2/7 of these patients had mastectomies). The latter might mean that both experienced readers have different strengths leading to an equal outcome. Hence, this might imply that getting better consensus about how to classify the tumors could still improve the results. This study highlights challenges and limitations in predicting response to NAC and determining residual disease in breast cancer.

### Comparison with other studies

In a recent study by Ballesio et al. (n = 51) the authors found that 65% of tumors with the concentric pattern halfway through NAC (n = 13; *p* < 0.001) had pCR, while none of the non-responders had the concentric pattern [14]. This is partly in agreement with our results. We found that 24% of the tumors with the type-1 pattern had pCR and 83% of tumors with the type-0 pattern which assumable together form the concentric pattern of their study. However, the percentage of non-responders with a type 1 pattern was 22% in our study. This difference may be due to the smaller number of patients (n = 51 versus n = 80) and the different choice of the MRI-based response patterns used. They only used three response patterns: concentric, nodular and mixed. This way they assumed all tumors shrink during NAC, while in our study 25% of tumors showed a stable response or progression. They also did not include a complete radiological response (i.e. pattern 0) while this was the group of patients with the strongest correlation with pCR in our study. We did not include a mixed pattern because the difference between “crumbling” and “crumbling and shrinking” might be minute and mostly subjective.

Two other studies studying MRI-based response patterns looked at MRI-based response patterns after NAC. The study of Golden et al. (only triple negative tumors, n = 60) had the same MRI-based response categories except they did not include diffuse enhancement (type 3) [15]. Even though our population only included 13 triple negative tumors, we found similar results in that MRI-based response patterns after NAC cannot successfully predict pathological outcome. Finally, our study was predominantly based on the study of Kim et al. (n = 55 (56 lesions)) [8], expanding and adjusting their classification. Like their results, concentric shrinking and crumbling tumors were more frequently observed in the pathological responder group. They more often noted the diffuse enhancing tumors in the non-responder group. As mentioned earlier, our number of diffuse enhancing tumors was too small to analyze. One of the most important additions in our study, as compared to their classification, was the definition of a complete response group (i.e. pattern 0), since this most accurately predicts pathological response. Furthermore, compared to their study, we tested the classification halfway through NAC in a larger cohort, showing stronger correlation to pathological response than after NAC.

One possible explanation for the better prediction of pathological response by MRI halfway through NAC than after NAC is that taxanes might suppress MRI enhancement irrespective of the cytotoxic activity. This finding was reported by Schrading et al. and since in our patient cohort taxanes were also only given during the second half of treatment, this could be a plausible explanation. Furthermore, as already shown by earlier studies, our study also shows that this might be false negative, especially when lobular carcinomas are classified as complete responders. Furthermore, the two triple negative tumors classified as type 0, were both true negatives.

### Strengths, limitations and future implications of this study

We studied the MRI patterns in the largest group of patients so far and only one comparable study was performed recently. Furthermore, this is the first study to look at interobserver agreement in MRI-based response patterns. The other studies that looked at MRI-based response patterns all performed a consensus reading of two radiologists. Therefore, none of them could test interobserver agreement. High interobserver agreement is important and desirable when we want to implement these MRI-based response patters in broad clinical practice. As mentioned earlier, we observed low interobserver agreement for the MRI-based response patterns. To increase interobserver agreement, a group of experienced radiologists should reach consensus about which tumors to classify under which MRI-based response pattern. Furthermore, it would also be desirable to reduce the interobserver differences in tumor diameter measurements. This for example could be done by looking into the strengths and weaknesses of experienced or even dedicated breast radiologists and making current practicing radiologists aware of these strong points and pitfalls.

One of the limitations of this study is that, as in the clinical setting, tumors were only measured on one slice on MRI and in one cutting direction by the pathologist. If the cutting direction was different from the MRI slice direction, the tumor diameter might differ. Another possible factor influencing diameter differences is that residual DCIS was excluded from the size estimation on pathological assessment, whereas this might have been visible on MRI, and therefore might have been included on the MRI evaluations. Furthermore, if multifocal disease was present, only the lesion with the largest dimensions on baseline MRI, considered to be the index tumor, was included for statistical analysis. Therefore, these results might not be applicable to multifocal tumors.

Future research should look into these MRI-based response patterns in an even larger group of patients with breast cancer. This way subgroup analyses can be performed to look at the differences in response patterns in the different subtypes of breast cancer. And last, we have to perform further research combining all response parameters (including subtypes, these MRI-based response patterns identified halfway through NAC and other parameters proven to help in response prediction, like MRI enhancement patterns and (semi-) automated tumor volume assessment) [16], to form a panel of biomarkers that enables individual response prediction.

## Conclusion

In patients with breast cancer undergoing NAC, tumor reduction > 50% was seen in about 70% of tumors and reduction in diameter > 50% was seen in about 40% of tumors. MRI-based response patterns halfway through NAC predicted pathological response more accurately than MRI-based response patterns after NAC. A complete radiological response halfway through NAC was associated with 83% pCR while a complete radiological response after NAC only seemed to be correct in 41% of the cases.

## Additional file

Additional file 1: **Appendix A.** Imaging parameters for standard breast MRI protocol. (DOCX 13 kb)

## Abbreviations

DCIS: Ductal carcinoma in situ
DWI: Diffusion weighted imaging
ER: Estrogen receptor
FISH: Fluorescence in situ hybridization
HER2: Human epidermal growth factor receptor 2
MUMC+: Maastricht University Medical Center
NAC: Neoadjuvant chemotherapy and immunotherapy
pCR: Pathological complete response
PR: Progesterone receptor
SPSS: Statistical Package for the Social Sciences
