# Supplementary material for: MRI-based response patterns during neoadjuvant chemotherapy can predict pathological (complete) response in patients with breast cancer
Source: Breast Cancer Res. 2018 Apr 18;20:34. doi: 10.1186/s13058-018-0950-x (PMC5907188; doi:10.1186/s13058-018-0950-x)
Supplement: Supplementary file 1 — Appendix A. Imaging parameters for standard breast MRI protocol. (DOCX 13 kb) [file 13058_2018_950_MOESM1_ESM.docx]

**Appendix A**. Imaging parameters for standard breast MRI protocol.

| Coil | Sense breast 16-channel |
| --- | --- |
| Matrix | 400 x 400 |
| Pixel spacing (mm) | 0.854^2^ |
| Field of View (mm) | 342^2^ |
| In-plane resolution (mm) | 0.9^2^ |
| Repetition time (msec) | 2000 |
| Echo time (msec) | 218 |
| Flip angle (°) | 90 |
| Echo train length | 95 |
| Slice thickness (mm) | 2.0 |
